# All Diesel Exhaust Is Not the Same: Engine Load Alters Toxicity

**DOI:** 10.1289/ehp.119-a355b

**Published:** 2011-08-01

**Authors:** Bob Weinhold

**Affiliations:** Bob Weinhold, MA, has covered environmental health issues for numerous outlets since 1996. He is a member of the Society of Environmental Journalists.

After decades of research on the adverse health effects of diesel engine exhaust (DEE), important information gaps still remain. Among these gaps are differences in the composition of DEE created when an engine operates under different load conditions (i.e., how hard the engine is working) as well as the health effects associated with various exhaust compositions. Some studies have evaluated exhaust products and health effects under one load condition or another, or with varying operating conditions during the same engine run cycle. But none have evaluated different load conditions in the same study, then looked at health effects caused by the separate exhaust products, according to the authors of a study that begins to address that knowledge gap [***EHP* 119(8):1136–1141; McDonald et al.**].

The team analyzed the composition of DEE generated from a single-cylinder diesel generator operating under full (100%) or partial (55%) load. They also evaluated several health end points in two strains of mice that inhaled the two resulting exhaust products, which varied substantially in composition.

The particulate matter (PM) concentrations in the exhaust products were similar to those found in certain occupational and high ambient outdoor settings. Under partial load, the PM constituent had higher organic carbon, ammonium, sulfate, and nitrate mass, lower elemental (black) carbon mass, and smaller particle size, compared with full-load exhaust. Vapor phase partial-load exhaust had a greater mass of carbon monoxide and nonmethane volatile organic compounds, a higher percentage of naphthalenes, and a lower percentage of alkanes.

**Figure:**
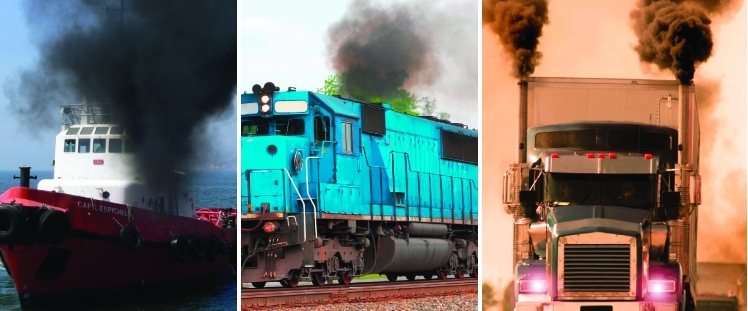
Results of a sturdy of a single-cylinder diesel generator operating under full and partial loads suggest not all diesel emissions are the same. Left to right: © Alan Smillie/Shutterstock; © Kenneth Sponsler/Shutterstock; © Mario Beauregard/Fotolia

Male C57B1/6 mice exposed to full-load exhaust had significantly more lung inflammation, as indicated through heme oxygenase-1 expression, compared with mice exposed to partial-load exhaust. These mice also demonstrated greater susceptibility to viral lung inflammation and epithelial damage and were much slower in clearing the virus from their lungs. However, mice exposed to partial-load exhaust had lower levels of two other inflammation indicators, interferon-γ and tumor necrosis factor-〈, than either control mice or those exposed to full-load exhaust.

In male ApoE^– / –^ mice, one measure of cardiovascular toxicity—reduction in heart rate—occurred significantly faster following exposure to partial-load exhaust compared with full-load exposure. Partial-load exhaust also was linked with a rapid increase in T-wave area, another indicator of cardiovascular toxicity, which was not affected by exposure to full-load exhaust.

The researchers conclude that the typical practice of evaluating DEE health effects based solely on the PM mass concentration of the exhaust is misleading. Instead, they recommend researchers carefully analyze and describe the DEE compositions they use in their studies so different bodies of work can be more accurately compared.

